# The Role of Minimally Invasive Adrenalectomy for Large Adrenal Tumors (≥6 cm): Evidence from a 10-Year Retrospective Study

**DOI:** 10.3390/jcm14155176

**Published:** 2025-07-22

**Authors:** Leonardo Rossi, Chiara Becucci, Ortensia Della Posta, Piermarco Papini, Francesca Palma, Mattia Cammarata, Luisa Sacco, Klaudiya Dekova, Suela Ajdini, Carlo Enrico Ambrosini, Gabriele Materazzi

**Affiliations:** Endocrine Surgery Unit, Department of Surgical, Medical and Molecular Pathology and Critical Area, University of Pisa, 56122 Pisa, Italy; bc.chibec@gmail.com (C.B.); o.dellaposta@studenti.unipi.it (O.D.P.); piermarcopapini@gmail.com (P.P.); palmesca@gmail.com (F.P.); mattiacamma@yahoo.it (M.C.); lois.sacco@gmail.com (L.S.); claudia.de003@gmail.com (K.D.); s.ajdini2@studenti.unipi.it (S.A.); carloeambrosini@gmail.com (C.E.A.); gabriele.materazzi@unipi.it (G.M.)

**Keywords:** adrenalectomy, laparoscopic adrenalectomy, retroperitoneoscopic, robotic adrenalectomy, large adrenal tumors, surgical outcomes

## Abstract

**Background**: The suitability of minimally invasive adrenalectomy (MIA) for adrenal tumors ≥6 cm remains debated due to technical challenges and oncological concerns. This study aimed to assess the safety and feasibility of MIA for large adrenal tumors by comparing surgical outcomes with smaller tumors. **Methods**: This retrospective cohort study included 269 patients who underwent MIA (2013–2023), divided into two groups: Group A (<6 cm, n = 197) and Group B (≥6 cm, n = 72). The primary endpoint was the postoperative complication rate; secondary endpoints included conversion to open surgery and postoperative length of stay (LOS). **Results**: Multivariate analysis identified no factors associated with postoperative complications; however, tumor size ≥ 6 cm was associated with conversion to open surgery (*p* = 0.031). Bilateral procedures and a higher Charlson comorbidity index were associated with longer LOS (*p* < 0.001 and *p* = 0.015, respectively). **Conclusions**: MIA is a safe and feasible approach for tumors ≥6 cm, despite being associated with a higher conversion rate.

## 1. Introduction

Currently, laparoscopic adrenalectomy (LA) is recognized as the standard treatment for small adrenal tumors, following its introduction by Gagner et al. [1] and Higashihara et al. [2] in 1992. The advantages of LA compared to open adrenalectomy include reduced postoperative pain, shorter hospital stays, and superior cosmetic outcomes [3,4,5,6,7,8,9,10]. Nowadays, the most commonly employed minimally invasive approaches for adrenalectomy are the lateral transabdominal (LTA) and the posterior retroperitoneal (PRA) adrenalectomies. The LTA offers a wider operative field, facilitates the identification of anatomical landmarks, and enables the simultaneous performance of other abdominal interventions, while the PRA is associated with a more direct route to the adrenal gland, the avoidance of intra-abdominal organ manipulation, and might be helpful in case of previous abdominal operations [11,12,13]. Moreover, in recent years, robot-assisted adrenalectomy has also gained increasing popularity, particularly due to its enhanced dexterity, three-dimensional visualization, and improved ergonomics, which may offer technical advantages in selected cases [8,14].

The suitability of minimally invasive adrenalectomy (MIA) for large adrenal tumors remains a subject of ongoing debate in the literature [14,15,16,17,18], primarily due to technical difficulties and oncological considerations. Historically, tumor size has been regarded as a key determinant of malignancy; consequently, open surgical management of malignant adrenal tumors has been preferred to minimize the risk of tumor spillage and capsule rupture [19,20]. Nonetheless, some authors argue that tumor size alone should not constitute an absolute contraindication for a minimally invasive approach [10,17].

There is no clear definition of the size of a large adrenal tumor [17]. However, the European Society of Endocrinology Clinical Practice Guidelines recommends laparoscopic surgery for adrenal tumors smaller than 6 cm without local invasion, while advocating an individualized surgical strategy for tumors exceeding 6 cm [21].

This study aims to compare the surgical outcomes of patients undergoing MIA for tumors ≥6 cm in size with those for tumors <6 cm.

## 2. Materials and Methods

### 2.1. Study Design and Population

This retrospective cohort study included all consecutive patients who underwent MIA at our Institution from January 2013 to December 2023. We included all patients undergoing MIA regardless of the surgical approach (laparoscopic or robotic LTA, PRA), lesion laterality, functionality, or histology, provided there was no radiological evidence of adjacent organ invasion. Patients who underwent open adrenalectomy (either for several previous laparotomic surgeries or for preoperative imaging suggestive of local infiltration) have been excluded.

**Figure 1:**
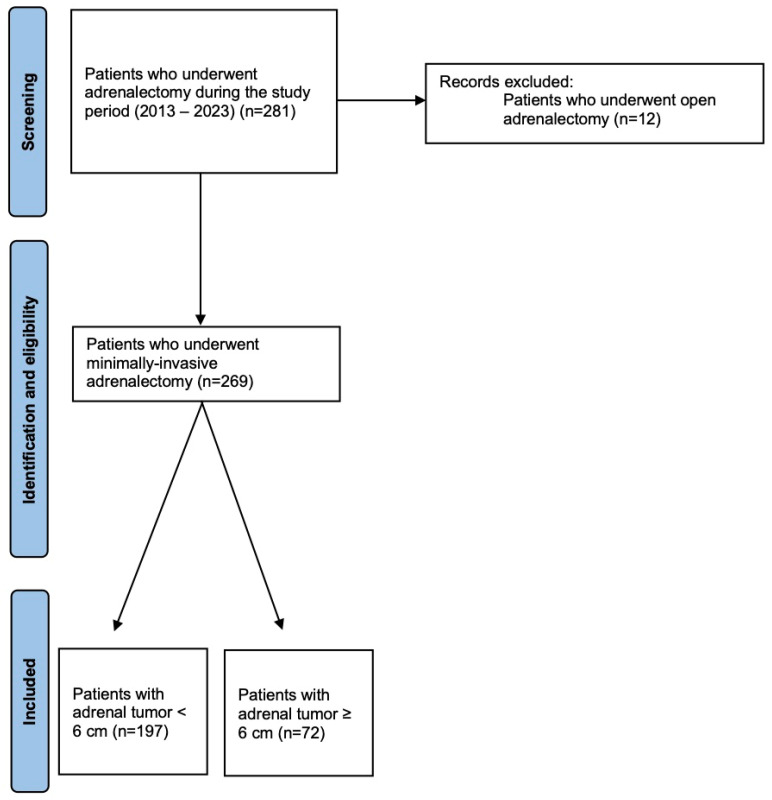
Study flowchart of patient enrollment.

A prospectively designed database was used for data collection and analysis. Patients were divided into two groups based on the tumor size: Group A included patients with a tumor <6 cm, while Group B included patients with a tumor ≥6 cm. A flowchart of enrolled patients is described in Figure 1.

Demographics and baseline patients’ characteristics included age, body mass index (BMI), gender, presence of comorbidities, prior abdominal surgery, hormonal hypersecretion, Charlson comorbidity index [22], American Society of Anesthesiologists (ASA) score [23], tumor location, surgical approach, associated surgeries, underlying genetic conditions, tumor size (in the case of bilateral surgery, the largest diameter was considered), and histologic diagnosis. For analysis, histologic diagnoses were categorized into cortical adenoma and cortical cyst, pheochromocytoma, myelolipoma, adrenal malignancies (including adrenal cortical carcinoma (ACC), angiosarcoma, and adrenal metastases), and others (including lymphoma, ganglioneuroma, angiomyolipoma, hemangioma, lymphangioma, and fibrous solitary tumor).

Moreover, we collected data regarding operative time (defined from the skin incision to the skin closure), conversion to open, reason for conversion, intraoperative complications, blood transfusion, postoperative length of hospital stay (defined as the days from the surgical operation to the discharge; LOS), postoperative complication rate, Clavien–Dindo Classification [24], Comprehensive Complication Index (CCI) [25], readmission at 30 days, and mortality.

The manuscript has been structured according to the Strengthening The Report Of Cohort Studies in Surgery (STROCCS) 2024 guideline [26].

The primary endpoint of this study was the rate of postoperative complications. Secondary endpoints were the conversion rate and the LOS.

This study has been approved by the Internal Review Board (IRB) (IRB code: 27924).

### 2.2. Surgical Techniques

#### 2.2.1. Lateral Transabdominal Adrenalectomy (Both Laparoscopic and Robotic)

**Figure 2:**
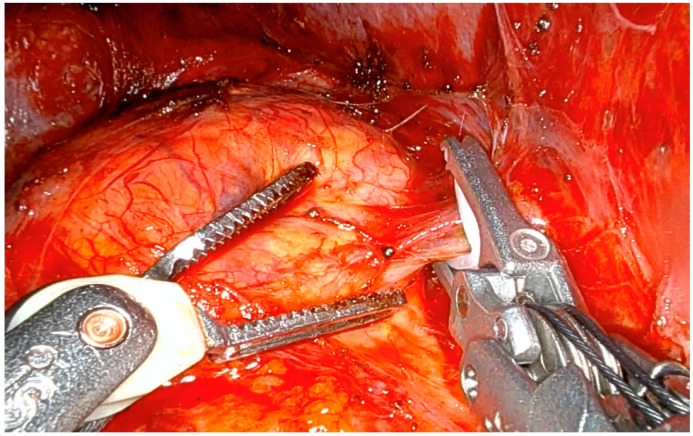
Clipping of the right middle adrenal vein during robotic LTA.

Both during laparoscopic and robotic LTA, patients were placed in a flank position (75–90°) with table flexion for optimal access. During laparoscopic LTA, three trocars were used for left and four for right adrenalectomy, with one additional trocar being used during the robotic counterpart. CO_2_ insufflation was maintained at 8–12 mmHg. On the left, the peritoneum was incised along the line of Toldt to mobilize the colon and spleen, enabling access to the adrenal gland. The middle adrenal vein, draining into the left renal vein, was clipped and transected before gland removal. On the right, liver mobilization exposed the retroperitoneum and inferior vena cava. The middle adrenal vein, draining into the inferior vena cava, was isolated, clipped, and divided (Figure 2), followed by gland dissection and removal.

#### 2.2.2. Posterior Retroperitoneoscopic Adrenalectomy

In the prone jackknife position, access to the retroperitoneum was achieved via a 1.5 cm incision below the 12th rib. After blunt dissection and positioning of the first two trocars, CO_2_ insufflation (20–30 mmHg) expanded the working space. The third trocar was placed under direct vision. Dissection started from the kidney’s upper pole to achieve adrenal resection. The middle adrenal vein was controlled near its confluence (inferior vena cava on the right, renal vein on the left). The specimen was retrieved in an endobag, and the workspace was irrigated and inspected to confirm hemostasis.

#### 2.2.3. Postoperative Course

All patients underwent early mobilization and resumed oral intake on postoperative day one, per our enhanced recovery protocol. Discharge occurred based on clinical status, typically within 1–3 days post-surgery.

### 2.3. Statistical Analysis

Continuous quantitative data were expressed as mean ± standard deviation (SD) and compared using Student’s *t*-test or Mann–Whitney U test, when appropriate. Categorical qualitative data were expressed as numbers and percentages and compared using the χ^2^ test (or Fisher’s exact test, when appropriate). Logistic and linear regression analyses were used to identify factors independently associated with postoperative complications, conversion to open surgery, and LOS. For linear and logistic regression modeling, tumor location was coded as a binary variable, with categories “unilateral” and “bilateral”. Factors with a *p*-value < 0.10 at univariate analysis were included in multivariate analysis models. Multivariable analyses were presented as odds ratios (ORs), when appropriate, coefficients, and *p*-values (95% confidence interval; CI).

All analyses were carried out with SPSS v.28 (IBM Corp., Armonk, NY, USA).

## 3. Results

### 3.1. Characteristics of the Study Population

Overall, 269 patients have been enrolled; in particular, 197 (73.2%) were included in Group A, while 72 (26.8%) were included in Group B. At univariate analysis, no difference between Group A and Group B was found in terms of age (52 ± 13.6 vs. 55.5 ± 13.5 years, respectively; *p* = 0.058), BMI (27.6 ± 6.8 vs. 27.9 ± 6.7 kg/m^2^, respectively; *p* = 0.795), presence of comorbidities (91.4% vs. 93.1%, respectively; *p* = 0.655), prior abdominal surgery (47.2% vs. 38.9%, respectively; *p* = 0.224), tumor location (*p* = 0.055), and underlying genetic mutation (6.1% vs. 9.7%, respectively; *p* = 0.303). On the other hand, at univariate analysis, a statistically significant difference was found in terms of gender (*p* = 0.018), Charlson comorbidity index (1.9 ± 1.8 vs. 2.4 ± 2.0, respectively; *p* = 0.049), ASA score (*p* < 0.001), hormonal hypersecretion (60.4% vs. 41.7%; *p* = 0.006), surgical approach (*p* < 0.001), histologic diagnosis (*p* < 0.001), and tumor size (3.6 ± 1.3 vs. 7.6 ± 1.8 cm, respectively; *p* < 0.001). These data are summarized in Table 1.

### 3.2. Intraoperative and Postoperative Outcomes

At univariate analysis, no statistically significant difference between Group A and Group B was found in terms of associated operations (11.7% vs. 9.7%, respectively; *p* = 0.652), conversion to open surgery (2.5% vs. 8.3%; *p* = 0.075), reason for conversion to open surgery (*p* = 0.143), intraoperative complications (1% vs. 2.8%, respectively; *p* = 0.646), blood transfusion (1% vs. 4.2%, respectively; *p* = 0.236), need for postoperative ICU (53.8% vs. 58.3%, respectively; *p* = 0.508), length of postoperative ICU stay (1 ± 0.2 vs. 1.1 ± 0.4 days, respectively; *p* = 0.406), LOS (3.1 ± 1.7 vs. 3.5 ± 2.3 days, respectively; *p* = 0.112), postoperative complications rate (9.6% vs. 11.1%, respectively; *p* = 0.723), Clavien–Dindo classification (*p* = 0.975), CCI (2 ± 6.7 vs. 2.1 ± 6.1, respectively; *p* = 0.857), and readmission at 30 days (2% vs. 1.4%, respectively; *p* = 0.730). A statistically significant difference was documented in terms of operative times between Group A and Group B (97.8 vs. 120 min, respectively; *p* = 0.002). These data are summarized in Table 2.

### 3.3. Diagnostic Workup and Oncological Outcomes of ACC Patients

In six (2.2%) patients, histological diagnosis revealed ACC, four (2%) included in Group A and two (2.8%) in Group B. All patients underwent a preoperative CT scan; two (33.3%) also underwent MRI, and one (16.7%) underwent MRI and PET scans. At preoperative imaging, five (83.3%) cases presented regular margins without a necrosis area, while one (16.7%) presented irregular margins with a heterogeneous intraparenchymal area consistent with necrosis. All cases presented without adjacent organ infiltration. ACC was incidentally identified through histological examination in all cases except one (16.7%), where a preoperative suspicion had already been raised due to high PET uptake and CT-scan features. The mean Hounsfield Unit of ACCs was 37. Three (50%) cases were non-functioning, whereas two (33%) exhibited cortisol hypersecretion and one (16.7%) demonstrated combined catecholamine and androgen hypersecretion. No capsular disruption was documented either intraoperatively or at histopathological examination. Moreover, an R0 resection was achieved in all cases.

Recurrence occurred in two (33.3%) patients, one per group, after a mean follow-up of 35 ± 21.1 months. The Group A ACC patient had recurrence in the retroperitoneum and ipsilateral kidney and is currently under treatment with mitotane with good disease control; conversely, the Group B ACC patient had recurrence in the retroperitoneum, treated surgically, and thereafter in the liver, treated by cryoablation, and the disease is controlled as of the last follow-up. The mean disease-free survival was 27 ± 14.8 months. No adrenal disease-related death occurred in the whole case series.

### 3.4. Multivariate Analyses

In logistic regression analysis, no factors were associated with postoperative complications (Table 3), while tumor size ≥ 6 cm was significantly associated with conversion to open surgery (*p* = 0.031) (Table 4).

In linear regression analysis, the Charlson comorbidity index and bilateral procedures were significantly associated with longer LOS (*p* = 0.015 and <0.001, respectively) (Table 5).

## 4. Discussion

Although it is well-known that MIA represents the best surgical option for small adrenal tumors, its role for large adrenal lesions is historically considered a debated topic. Nonetheless, the cut-off of 6 cm is not based on good clinical evidence [21]. The primary challenge lies in the need for extensive tumor manipulation during surgery and difficulties in tumor handling. Furthermore, tumor size has traditionally been considered a critical factor in estimating malignancy, with the prevailing belief that malignant adrenal tumors are best managed through open surgery to reduce the risk of tumor spillage and capsule rupture [19,20]. However, these concerns have been progressively addressed over time. Advances in perioperative management, a deeper understanding of adrenal disease pathophysiology, and significant improvements in laparoscopic techniques, particularly those enhancing visualization and minimizing gland manipulation, have rendered tumor size no longer an absolute contraindication to laparoscopic surgery in appropriately selected patients [13,27,28].

In the present study, a 10-year experience involving 269 patients undergoing adrenalectomy via a minimally invasive approach is reported. The two groups include a wide range of preoperative diagnoses. Comparable outcomes were observed in terms of safety for adrenal tumors <6 or ≥6 cm, with no difference regarding intra- and postoperative complications. These findings support the reliability of MIA for large adrenal masses when performed in experienced settings. These results are consistent with previous studies documenting the safety of MIA regardless of tumor size when performed by skilled surgeons [13,29,30,31]. Conversely, some authors [28,32,33] documented a higher rate of postoperative complications for large adrenal tumors, although they reported that these are generally low and that 6 cm should not be considered the upper limit for performing MIA in high-volume institutions.

An assessment between groups was conducted, irrespective of the specific minimally invasive approach employed (prone retroperitoneoscopic, transabdominal lateral laparoscopic or robotic). It is of paramount importance to emphasize that a tailored surgical approach should be adopted based on a case-by-case assessment. In the present series, a marked predominance of the lateral transabdominal approach was observed for large adrenal tumors, with over 95% of cases managed via this route. This finding is consistent with that reported by Mihai et al. [34] and may be attributed to the familiar anatomical orientation and broader operative field offered by this technique, which can facilitate the handling of large adrenal masses. Nevertheless, the choice of surgical approach is largely influenced by the surgeon’s expertise and preference. Walz et al. demonstrated that PRA can be a suitable option for adrenal lesions up to 8 cm in diameter [11]. In recent years, robotic adrenalectomy has also shown promising results in the management of large adrenal tumors, owing to its inherent advantages such as enhanced dexterity, articulated instruments, and three-dimensional visualization [8]. Therefore, a comprehensive evaluation of patient- and tumor-specific factors is essential in selecting the most appropriate surgical approach. Ideally, all these techniques should be part of the surgeon’s operative repertoire. Nevertheless, in the present study, no association was observed between the surgical approach and postoperative complications, conversion to open surgery, or prolonged LOS.

A higher rate of conversion to open surgery was associated with large adrenal tumors in multivariate analysis. This is likely due to difficulties in handling large adrenal masses and their higher likelihood of bleeding, but these issues can be considered regardless of the surgical approach [13,18]. Therefore, it appears questionable to systematically refer large adrenal masses to open surgery since conventional adrenalectomy is not easier than MIA. Moreover, the conversion rate for large adrenal masses was relatively low (8.3%), in line with previous reports (6.7–14.6%) [13,18,32]. Notably, four out of six conversions in Group B occurred in right-sided adrenal tumors; this may be due to the retrocaval growth of large adrenal masses, which may have made the dissection challenging.

A longer operative time was documented for adrenal lesions ≥6 cm, in agreement with the existing literature [13,18,32]. This is likely related to more demanding dissection of large adrenal masses, which may dislocate surrounding structures. Tiberio et al. reported a significant increase in intraoperative complications and hospital stay for adrenalectomies lasting more than 140 min [35]. However, the mean operative time in both groups was below the threshold reported by Tiberio et al. [35], and we hypothesized that our difference in operative time between groups, although statistically significant, may be clinically negligible. Additionally, approximately 10% of patients in each group underwent associated procedures along with adrenalectomy, prolonging the overall duration of the intervention.

No association was observed between large adrenal masses and prolonged LOS, confirming previous findings [13,28,36]. In contrast, the Charlson comorbidity index and bilateral procedures were associated with longer LOS. These findings appear intuitive, as patients with greater comorbidities and those who experienced iatrogenic adrenal insufficiency are usually associated with longer hospitalization [33].

The main concern regarding the minimally invasive treatment of adrenal lesions is the increasing risk of malignancy with size. The NIH consensus statement reported that the rate of incidental ACC is 2% for lesions smaller than 4 cm, 6% for tumors sized between 4 and 6 cm, and 25% for tumors larger than 6 cm [37]. Therefore, there are controversies in handling large adrenal masses due to the fear of tumor capsule disruption and tumor spillage, leading to tumor recurrence. Nonetheless, these complications may occur even during open adrenalectomy. Thus, the surgical approach should not be considered a contraindication; rather, the surgeon’s skills determine the safety of the resection. Moreover, an accurate preoperative workup may raise the suspicion of adrenal cortical carcinoma, either for the radiological characteristics of the tumor itself or for the presence of organ invasiveness or lymph node metastasis. Furthermore, intraoperative findings, potentially aided even by intraoperative ultrasound [38,39], could inform surgeons regarding the suspicion of a malignant lesion or guide resection for precise surgical margins. The present study did not specifically investigate the oncological safety of MIA for malignant adrenal lesions and its comparison with open adrenalectomy. Nevertheless, the incidence of ACC in Group B was less than 3%; thus, opting for an open approach for all adrenal masses larger than 6 cm would preclude the vast majority of patients from the benefits of MIA, in particular, faster recovery, less estimated blood loss, and shorter LOS [40]. In the present cohort, all ACC cases included showed no radiological or intraoperative signs of adjacent organ invasion. This was a key determinant in selecting the surgical approach, as per current guidelines [21], which recommend open adrenalectomy in cases with suspected local infiltration. Therefore, following our study design, patients with signs of local invasion were excluded and managed with open surgery, whereas the minimally invasive approach was reserved for cases without evidence of infiltration. Overall, six cases of ACC were observed, four with tumors sized <6 cm and two sized ≥6 cm. All but one diagnosis were incidental, and surgery without capsule disruption led to an R0 resection in all cases. Nonetheless, recurrence occurred in two patients, one included in Group A (local relapse) and one in Group B (local relapse and liver metastasis). After a mean follow-up of 35 months, all patients are still alive. Although the topic remains controversial [19,20,41], Machado et al. reported in a systematic review that there was no difference in terms of local recurrence, positive resection margins, peritoneal carcinomatosis, and time to recurrence between laparoscopic and open adrenalectomy. The authors concluded that a poor outcome is more likely related to inadequate surgery rather than the chosen approach [42]. Similar findings are reported by other authors [27,43,44].

The main limitation of the present study is its retrospective design, which may have introduced selection bias. Additionally, the cohort includes patients with heterogeneous adrenal histologies treated by several different minimally invasive approaches and lacks a specific oncological comparison with open adrenalectomy in patients with ACC. Another limitation of the present study is the imbalance in group sizes, with a smaller number of patients in the ≥6 cm group. Although a formal sample size calculation is not typically applicable to retrospective studies, the current sample size may not be sufficient to identify smaller, yet potentially clinically relevant, differences in postoperative outcomes. This aspect should be taken into account when interpreting the findings, and future larger multicenter studies are warranted to confirm our results. Finally, as this study reflects the experience of a high-volume center, the findings may not be generalizable to other settings.

## 5. Conclusions

In conclusion, MIA for large adrenal tumors (≥6 cm) is safe and technically feasible in experienced hands, although it is associated with longer operative time and a higher conversion rate. Nonetheless, these findings should not overshadow the advantages of MIA for these patients.

While the oncological safety of MIA for confirmed or suspected ACC remains a matter of ongoing investigation, our experience suggests that, in the absence of radiological signs of local invasion, MIA may still be performed safely for large adrenal masses, regardless of tumor size. Given the low incidence of ACC in lesions ≥6 cm in our selected cohort, adopting an open approach for all large adrenal tumors would unnecessarily deprive most patients of the well-established benefits of MIA. However, careful patient selection and referral to a high-volume institution with extensive expertise in adrenal tumor management are key to achieving optimal outcomes.

## Figures and Tables

**Table 1:** Univariate analysis of baseline, preoperative, and histologic characteristics of the study groups.

Parameter	Group A (<6 cm) N = 197 (73.2%)	Group B (≥6 cm) N = 72 (26.8%)	*p* Value
Age, mean ± SD (years)	52 ± 13.6	55.5 ± 13.5	0.058
BMI, mean ± SD (kg/m^2^)	27.6 ± 6.8	27.9 ± 6.7	0.795
Female, n. (%)	75 (38.1)	38 (54.2)	0.018
Male, n. (%)	123 (61.9)	41 (45.8)	
Comorbidity, n. (%)	180 (91.4)	67 (93.1)	0.655
Charlson comorbidity index, mean ± SD	1.9 ± 1.8	2.4 ± 2.0	0.049
ASA score, n. (%)			<0.001
1	1 (0.5)	5 (6.9)	
2	113 (56.3)	26 (34.7)	
3	82 (42.1)	48 (58.3)	
4	2 (1)	0 (0)	
Prior abdominal surgery, n. (%)	93 (47.2)	28 (38.9)	0.224
Hormonal hypersecretion, n (%)	131 (66.5)	30 (41.7)	<0.001
Catecholamine, n (%)	51 (25.9)	19 (26.4)	
Aldosterone, n (%)	38 (19.3)	2 (2.8)	
Cortisol, n (%)	41 (20.8)	9 (12.5)	
Cortisol + Aldosterone, n (%)	1 (0.5)	0	
Catecholamine + Androgen, n (%)	0	1 (1.4)	
Surgical approach			<0.001
Laparoscopic LTA, n. (%)	149 (75.6)	69 (95.8)	
PRA, n. (%)	43 (21.8)	1 (1.4)	
Robotic LTA, n. (%)	5 (2.5)	2 (2.8)	
Tumor location:			0.055
Right, n. (%)	95 (48.2)	45 (62.5)	
Left, n. (%)	95 (48.2)	23 (31.9)	
Bilateral, n. (%)	7 (3.6)	4 (5.6)	
Genetic mutation, n. (%)	12 (6.1)	7 (9.7)	0.303
21-OHD, n. (%)	0 (0)	1 (1.4)	
MEN2A, n. (%)	5 (2.5)	4 (5.6)	
MEN2B, n. (%)	2 (1)	0 (0)	
NF1, n. (%)	3 (1.5)	1 (1.4)	
VHL, n. (%)	2 (1)	0 (0)	
Maffucci syndrome, n. (%)	0 (0)	1 (1.4)	
Tumor size (major lesion), mean ± SD (cm)	3.6 ± 1.3	7.6 ± 1.8	<0.001
Histology			<0.001
Cortical adenoma or cyst, n. (%)	131 (66.5)	28 (38.9)	
With AMH, n (%)	6 (4.6)	2 (2.8)	
Pheochromocytoma, n. (%)	45 (22.8)	17 (23.6)	
Adrenal malignancies, n. (%)	10 (5.1)	9 (12.5)	
ACC, n.(%)	4 (2)	2 (2.8)	
Angiosarcoma, n. (%)	0	1 (1.4)	
Adrenal metastasis, n. (%)	6 (3)	6 (8.3)	
Myelolipoma, n. (%)	1 (0.5)	14 (19.4)	
Others, n. (%)	10 (5.1)	4 (5.6)	
Lymphoma, n. (%)	0	2 (2.8)	
Ganglioneuroma, n. (%)	3 (1.5)	2 (2.8)	
Angiomyolipoma, n. (%)	1 (0.5)	0	
Hemangioma, n. (%)	1 (0.5)	0	
Lymphangioma, n. (%)	3 (1.5)	0	
Fibrous solitary tumor, n. (%)	2 (1)	0	

SD: standard deviation; ASA: American Society of Anesthesiologists; BMI: body mass index; 21-OHD: 21-hydroxylase deficiency; MEN2A: multiple endocrine neoplasia type 2A; MEN2B: multiple endocrine neoplasia type 2B; NF1: neurofibromatosis type 1; VHL: von Hippel–Lindau disease; ACC: adrenal cortical carcinoma; AMH: adrenal medullary hyperplasia.

**Table 2:** Univariate analysis of operative and postoperative outcomes of the study groups.

Parameter	Group A (<6 cm) N = 197 (73.2%)	Group B (≥6 cm) N = 72 (28.8%)	*p* Value
Operative time, mean ± SD (min)	97.8 ± 50.5	120 ± 56.2	0.002
Associated surgeries, n. (%)	23 (11.7)	7 (9.7)	0.652
Conversion to open, n. (%)	5 (2.5)	6 (8.3)	0.075
Reason for conversion			0.143
Technical difficulties, n. (%)	1 (0.5)	4 (5.6)	
Hemodynamic instability, n. (%)	0 (0)	2 (2.8)	
Others, n. (%)	4 (2)	0 (0)	
Intraoperative complications	2 (1%)	2 (2.8%)	0.646
No complications, n. (%)	195 (99.0)	70 (97.2)	
Hemorrhage, n. (%)	0 (0)	1 (1.4)	
Iatrogenic damage, n. (%)	1 (0.5)	0 (0)	
Others, n. (%)	1 (0.5)	1 (1.4)	
Blood transfusions, n. (%)	2 (1.0)	3 (4.2)	0.236
Postoperative ICU, n. (%)	106 (53.8)	42 (58.3)	0.508
Length of ICU stay, mean ± SD (days)	1 ± 0.2	1.1 ± 0.4	0.406
Length of hospital stay, mean ± SD (days)	3.1 ± 1.7	3.5 ± 2.3	0.112
Postoperative complications, n. (%)	19 (9.6)	8 (11.1)	0.723
Clavien–Dindo classification			0.975
Grade 1, n. (%)	6 (3)	2 (2.8)	
Grade 2, n. (%)	10 (5.1)	6 (8.3)	
Grade 3a, n. (%)	1 (0.5)	0	
Grade 4a, n. (%)	2 (1)	0	
Comprehensive Complications Index, mean ± SD	2.0 ± 6.7	2.1 ± 6.1	0.857
Readmission at 30 days, n. (%)	4 (2)	1 (1.4)	0.730
Adrenal-related mortality, n (%)	0	0	NA

SD: standard deviation; ICU: intensive care unit; NA: not available.

**Table 3:** Logistic regression analysis of factors associated with postoperative complications.

Parameter	Coefficient	*p*-Value	Odds Ratio	95% CI	
				Inferior	Superior
Tumor size ≥ 6 cm	0.149	0.768	1.161	0.432	3.117
Age	−0.027	0.197	0.973	0.934	1.014
Gender	0.091	0.838	0.745	0.455	2.637
ASA score	0.525	0.240	1.691	0.704	4.063
Charlson comorbidity index	0.282	0.056	1.326	0.993	1.771
Hormonal hypersecretion	0.378	0.426	1.459	0.575	3.701
Tumor location (unilateral or bilateral)	−0.223	0.851	0.800	0.078	8.162
Surgical approach	−0.064	0.841	0.938	0.503	1.749
Histology	0.091	0.658	1.096	0.731	1.642

ASA: American Society of Anesthesiologists; CI: confidence interval.

**Table 4:** Logistic regression analysis of factors associated with conversion rate.

Parameter	Coefficient	*p*-Value	Odds Ratio	95% CI	
				Inferior	Superior
Tumor size ≥ 6 cm	1.758	0.031	5.800	1.177	28.595
Age	0.008	0.842	1.008	0.928	1.096
Gender	0.855	0.266	2.351	0.522	10.587
ASA score	1.662	0.057	5.271	0.950	29.253
Charlson comorbidity index	0.022	0.930	1.023	0.620	1.688
Hormonal hypersecretion	−0.388	0.632	0.678	0.138	3.327
Tumor location (unilateral or bilateral)	0.853	0.526	2.346	0.168	32.808
Surgical approach	0.184	0.772	1.202	0.347	4.157
Histology	0.029	0.927	1.030	0.548	1.935

ASA: American Society of Anesthesiologists; CI: confidence interval.

**Table 5:** Linear regression analysis of factors associated with LOS.

Parameter	Coefficient	*p*-Value	95% CI	
			Inferior	Superior
Tumor size ≥ 6 cm	0.248	0.344	−0.268	0.764
Age	−0.011	0.332	−0.034	0.011
Gender	0.203	0.378	−0.251	0.657
ASA score	0.416	0.062	−0.021	0.853
Charlson comorbidity index	0.211	0.015	0.041	0.381
Hormonal hypersecretion	−0.276	0.239	−0.737	0.185
Tumor location (unilateral or bilateral)	3.231	<0.001	2.037	4.424
Surgical approach	−0.193	0.207	−0.494	0.108
Histology	0.051	0.630	−0.157	0.259

ASA: American Society of Anesthesiologists; CI: confidence interval.

## Data Availability

The data that support the findings of this study are available from the corresponding author upon reasonable request.
